# Analysis of outcomes of endovascular embolisation: A cross-sectional two-center study on 46 visceral artery pseudoaneurysms

**DOI:** 10.1186/s42155-021-00248-0

**Published:** 2021-07-16

**Authors:** Mohammad Koriem Mahmoud Omar, Moustafa H M Othman, Robert Morgan, Abdelkarem Hasan Abdallah, Hany Seif, Mohamed Zidan, Mahmoud Khairallah, Reham Abd El-Aleem

**Affiliations:** 1 Department of Diagnostic and Interventional Radiology, Assiut University Hospitals, Assiut, Egypt; 2 Department of Interventional Radiology, St. George’s University Hospitals NHS Foundation Trust, London, UK

**Keywords:** Endovascular, Embolisation, Coils, NBCA, Glue, Visceral, Pseudoaneurysms

## Abstract

**Purpose:**

Visceral artery pseudoaneurysms (VAPAs) are uncommon in clinical practice but may have serious clinical outcomes up to death. Endovascular management is a safe effective alternative option to traditional surgical procedures. This study assesses the outcome of different embolic materials and techniques used in the endovascular management of VAPAs.

**Materials and methods:**

This is a two-center retrospective analysis of endovascular embolisation of 46 VAPAs, with a mean pseudoaneurysm size of 13 ± 11.35 mm, that were urgently managed between July 2018 and March 2020. Patients’ presentations were GIT hemorrhage, intrabdominal hemorrhage, hematuria, and abdominal pain in 34.78%, 30.43%, 23.91%, and 10.87% respectively. Management using coils only was done in 28/46 patients (60.87%), NBCA glue only in 16/46 patients (34.78%), combined coils and NBCA glue in 1/46 patient (2.17%), and Amplatzer plugs only in 1 patient (2.17%). The management techniques were sac packing in 9/46 patients (19.57%), inflow occlusion in 28/46 patients (60.87%) and trapping in 9/46 patients (19.57%). All patients were followed-up for 1 year after the procedure.

**Results:**

The overall clinical success and periprocedural complication rates were 93.48%, and 15.22% respectively, and 30-day mortality was zero. Clinical success was 92.86% in the coil subgroup (*n* = 28), and 93.75% in the NBCA glue subgroup (*n* = 16). The technical success rate was 100%. Effectiveness of the procedures during the follow-up was 97.83%. Target lesion re-intervention rate was 2.17%.

**Conclusion:**

Transarterial embolisation can provide high technical and clinical success rates with low periprocedural complication and re-intervention rates, as well as satisfactory procedure effectiveness in the management of VAPAs.

## Background

Visceral artery aneurysms (VAAs) typically occur within celiac trunk and its branches, superior or inferior mesenteric arteries and renal arteries. Unlike true aneurysms that are localized dilatation of the artery with the involvement of all arterial wall layers, pseudoaneurysms are effectively contained ruptures of the artery that are lined by adventitia or by the perivascular tissues (Belli et al. 2012). Generally, true aneurysms are asymptomatic and occur secondary to underlying arterial diseases while pseudoaneurysms are a sequence of direct trauma, or inflammation of the vessel (Belli et al. 2012). Despite the rarity of true visceral artery aneurysms, pseudoaneurysms are more frequently encountered in specialized centers dealing with acute trauma patients or high volumes of abdominal interventions than true aneurysms that are often incidentally discovered (Jesinger et al. 2013).

The imaging appearance of visceral artery pseudoaneurysms (VAPAs) is similar to that of true aneurysms, but typically exhibit more irregular margins, and the pseudoaneurysm is typically surrounded by a hematoma (Jesinger et al. 2013). Up to 70% of pseudoaneurysms and 20% of true aneurysms are liable to rupture and mortality occurs in 25% to 100% (Pitton et al. 2015). Hyperdynamic circulation e.g. pregnancy, portal hypertension and infections are risk factors for rupture. Eighty per cent of the aneurysms of the hepatic artery are liable to rupture, followed by aneurysms of SMA and pancreaticoduodenal arcades (Bradley et al. 2019; van Rijn et al. 2017; Durkin et al. 2016).

In general, asymptomatic true visceral artery aneurysms that are less than 2 cm can be followed up without further management (Jesinger et al. 2013; Madhusudhan et al. 2016). On the other hand, pseudoaneurysms must be managed regardless their presentation, size and location owing to their high possibility of rupture (Madhusudhan et al. 2016).

Endovascular management of VAPAs has been widely used as a safe and effective alternative treatment to the more invasive surgical procedures with higher mortality rate reaching 5% and mortality increases substantially if emergency surgery is required for aneurysm rupture repair (Loffroy 2015; Cappucci et al. 2017; Venturini et al. 2017; Martinelli et al. 2019). Endovascular treatment options include techniques for parent artery preservation: sac packing with embolic materials e.g. (coils, or glue e.g. onyx and N-butylcyanoacrylate), stent graft exclusion, balloon remodeling or stent-assisted coiling and parent artery sacrifice techniques: trapping or inflow occlusion (Madhusudhan et al. 2016). The current study was designed to review the outcome of different embolic materials and techniques used in the endovascular management of visceral artery pseudoaneurysms. True visceral artery aneurysms are uncovered in this article.

## Methods

This is a two-center retrospective analysis of 46 patients with 46 VAPAs of any size who had urgent endovascular management between July 2018 and March 2020. Informing consent of participant was not necessary as the study is retrospective. Those patients were presenting with either abdominal pain, or intrabdominal hemorrhage, or gastrointestinal (GIT) bleeding and/ or hemobilia or hematuria. A full medical history of co-morbidities and risk factors was taken for each patient. Clinical assessment and abdominal ultrasonography were done to all patients. Hemodynamically unstable patients received urgent medical support before further assessment.

Computed tomography angiography (CTA) was done to diagnose and confirm VAPAs in all patients prior to catheter angiography. CTA was performed either with a 64-slice multidetector helical CT, the Siemens SOMATOM Sensation 64 or 128-slice multidetector helical CT, the Siemens SOMATOMS Definition 128 (Siemens, Erlangen, Germany).

The following data were recorded: age, sex, associated co-morbidities along with risk factors, presentation, size as well as shape of pseudoaneurysm, affected artery, and location of the lesion within the artery (proximal, middle, or distal).

### Endovascular embolisation technique

Under local anesthesia, the procedures were performed by experienced (> 10 years) interventional radiologists in dedicated interventional radiology suites on Artis Zee flat-type monoplane or Artis Q biplane digital subtraction angiography machines (Axiom-Artis; Siemens, Erlangen, Germany). Right transfemoral artery approach was performed in all cases.

Arterial access to the lesions was achieved by using 4 or 5 Fr standard angiographic catheters (Cobra, C1 angiographic catheter; Cook; Bloomington, IN), or (Sidewinder Simmons, Sim 1 Cordis; Johnson & Johnsons, Miami, FL) and 2.4 or 2.7 Fr coaxial microcatheter (Progreat Terumo Corporation, Tokyo, Japan) with different guide wires. The decision to use a certain embolisation technique and different types of embolic materials or even a combination was operator dependent and based on the arterial anatomy and on the availability of the materials in the emergency settings. Embolisation using coils only was done in 28/46 patients, while N-butylcyanoacrylate (NBCA) glue only was used in 16/46 patients. Combined coils and NBCA glue were used in 1/46 patient, and Amplatzer vascular plugs were used in 1 patient.

When embolisation was performed using metallic detachable or pushable coils [MReye (Cook) or Interlock (Boston Scientific)] of variable diameters and lengths; the coils were oversized by ~ 20% compared with the target artery diameter.

When NBCA glue (Histoacryl Blue®; B. Braun, Melgungen, Germany) was used, the tip of the microcatheter was placed inside the aneurysm sac or as close as possible to the neck of the aneurysm. However, if the catheter tip could not be properly placed at the neck of the aneurysm because of the small caliber or tortuosity of the artery, it was wedged into the inlet of the arteries to be embolised to limit retrograde pericatheter reflux of the glue.

According to the desired rate of polymerization, NBCA was diluted manually with ethiodized oil (Lipiodol Ultra-Fluid®; Guerbet, Roissy-Charles-de-Gaulle, France), a polymerization-retardant. Specifically, when embolising a vessel of high-rate blood flow, or when the catheter was intralesional, a quick in vivo polymerization was required and a ratio of 1:1 oil to NBCA was used. To delay glue polymerization, in situations where the microcatheter tip was positioned distant from the desired site of polymerization, a greater volume of ethiodized oil (ie, 2:1, 3:1 dilutions) was added.

The lumen of the microcatheter was flushed with 5% dextrose before injection of the NBCA mixture, thus preventing polymerization before reaching the arterial segments. Using a 1-mL syringe and under careful fluoroscopic monitoring, NBCA mixture was injected. In order to prevent adherence of the catheter tip to the vessel wall, the microcatheter was removed immediately after injection. Then, the guiding catheter was aspirated to clear its inner lumen, and post-embolic angiography was performed.

**Fig. 1:**
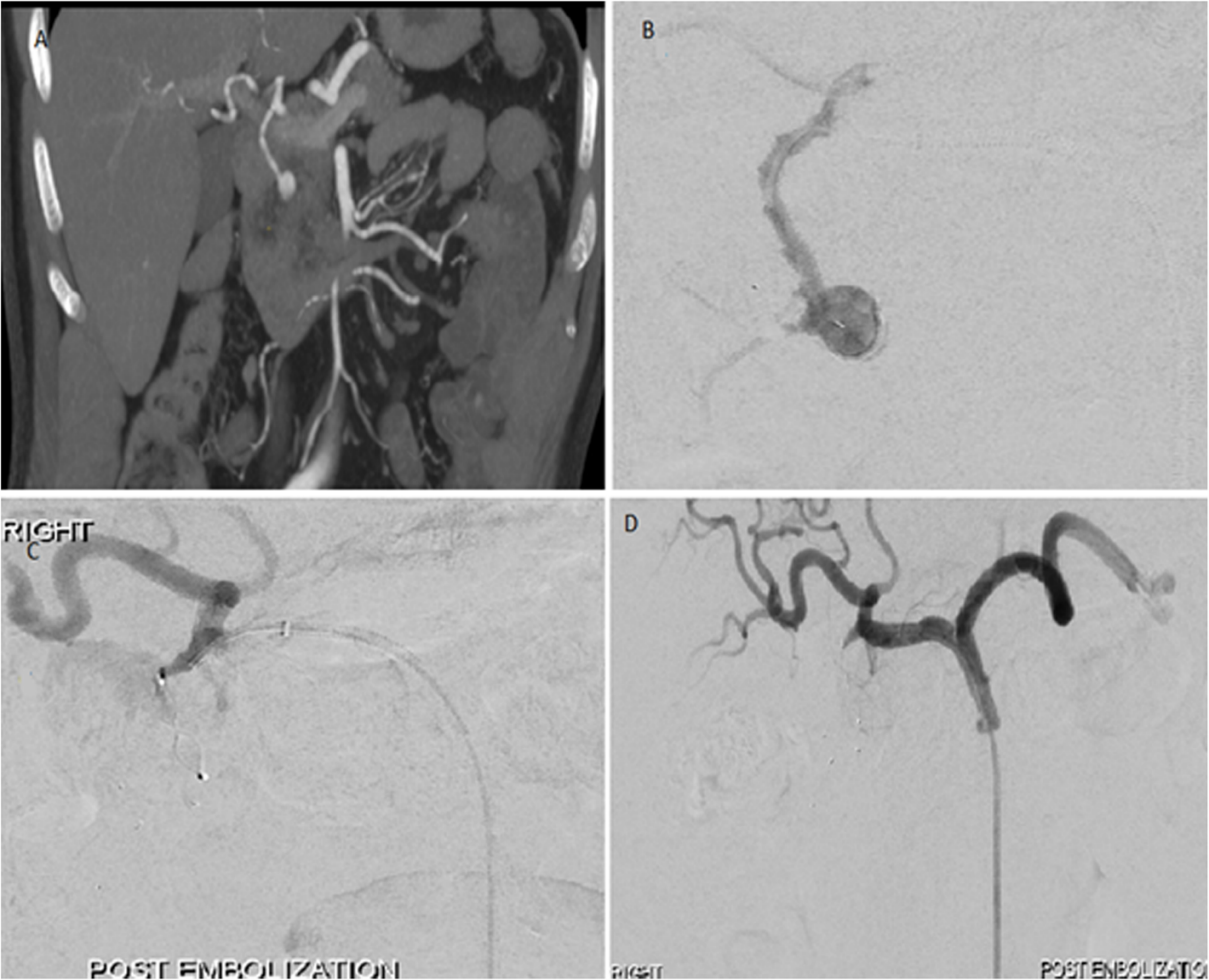
Embolisation of gastroduodenal artery pseudoaneurysm. **a**. CT angiogram showing gastroduodenal artery pseudoaneurysm surrounded by hematoma. **b** Selective angiogram of the gastroduodenal artery demonstrating the lesion. **c** Embolisation of the gastroduodenal artery pseudoaneurysm by trapping technique with 6.5 mm and 5 mm diameter microvascular plugs distally & 7 mm diameter Amplatzer IV plug proximally. **d** Final angiogram showing complete exclusion of the pseudoaneurysm from the circulation

Amplatzer Vascular Plugs (St Jude Medical, St Paul, MN, USA) were used in a selected case (Fig. 1) where there was a pseudoaneurysm in a high-flow gastroduodenal artery (GDA) in order to reduce the risk of migration and systemic embolisation of traditional occlusion devices.

**Fig. 2:**
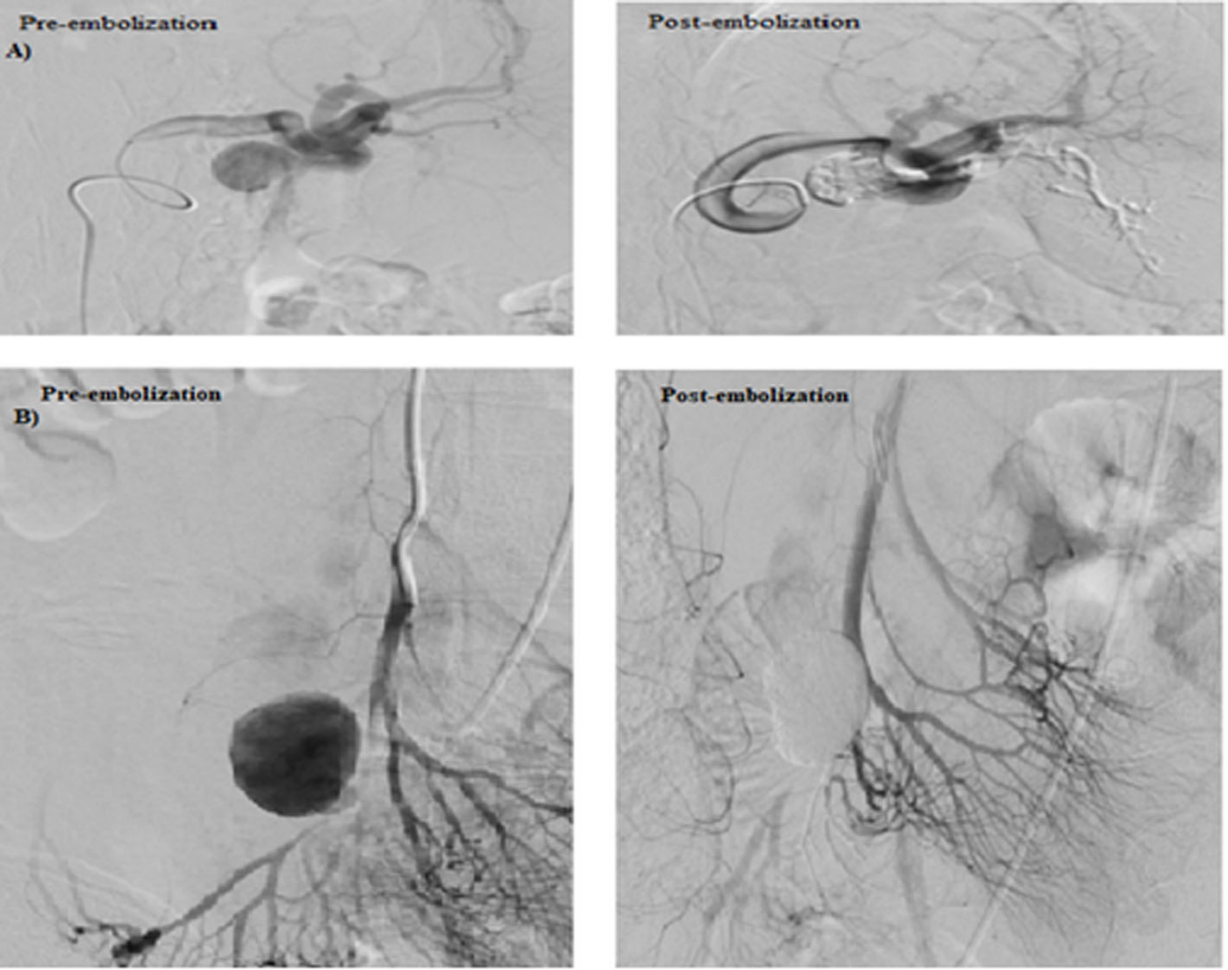
Sac packing embolization technique with NBCA/ Lipiodol mixture. **a** Embolisation of splenic artery pseudoaneurysm by sac packing technique with NBCA/ Lipiodol mixture with non-significant distal spillage of the embolic material in the lower pole branch of the splenic artery. **b** Embolisation of superior mesenteric artery (SMA) pseudoaneurysm by sac packing technique with NBCA/ Lipiodol mixture with spillage of the embolic material distally. This distal spillage was non-significant owing to the good collateral circulation

**Fig. 3:**
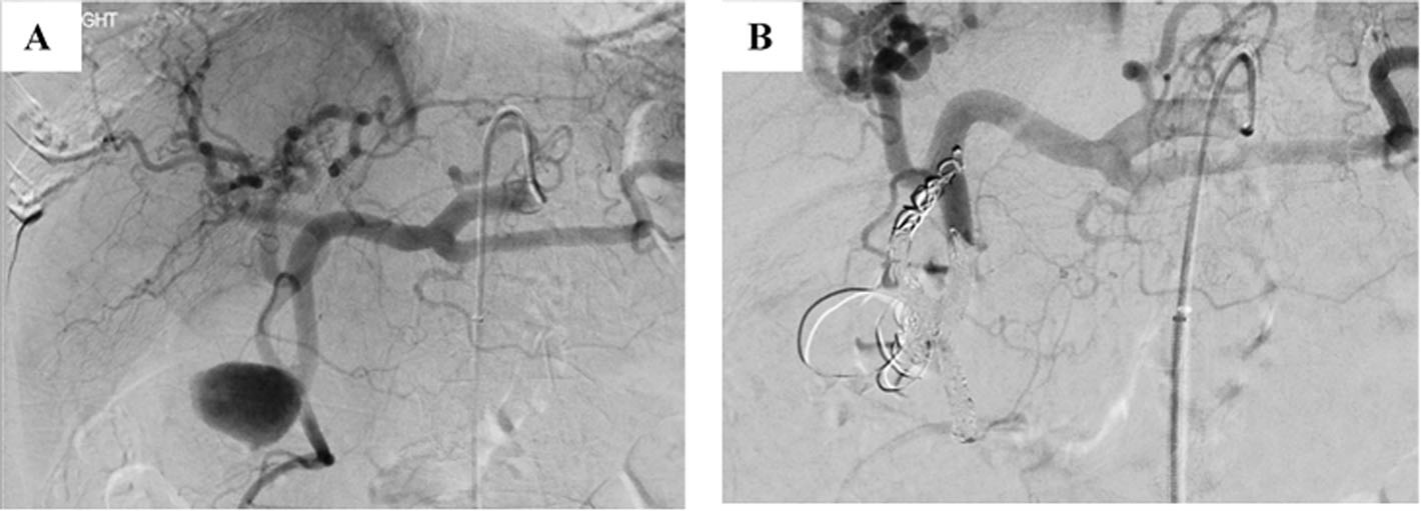
Embolisation of gastroduodenal artery pseudoaneurysm by trapping technique with multiple micro coils. **a** Selective angiogram of the celiac axis and gastroduodenal artery demonstrate pseudoaneurysm arising from the gastroduodenal artery with associated replaced right hepatic artery arising from the gastroduodenal artery at the neck of the pseudoaneurysm. **b** Embolisation of the front and back doors of the pseudoaneurysm as well as the replaced right hepatic artery using 4, 5 and 6 mm detachable 0.018 coils

**Fig. 4:**
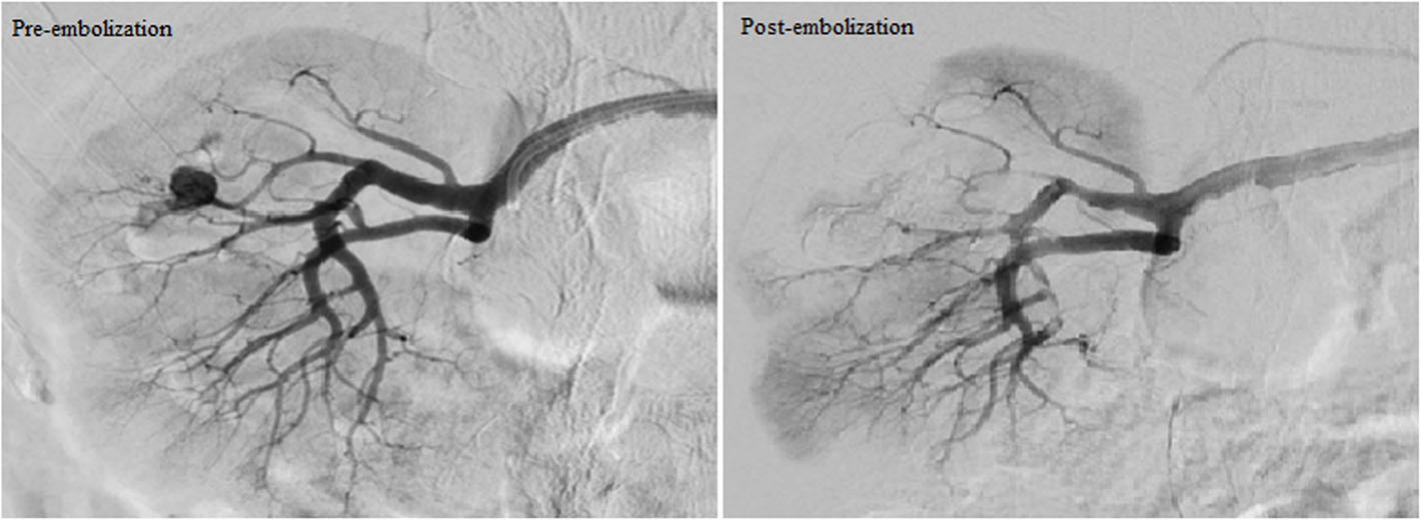
Embolisation of right renal artery pseudoaneurysm by inflow occlusion technique with NBCA/ Lipiodol mixture

The embolisation techniques used in the study are illustrated in Table 1. Figures 2, 3 & 4 show the use of different embolic materials and techniques in the management of different visceral artery pseudoaneurysms.

**Table 1:** Endovascular embolisation techniques used in the study

**Parent vessel flow preservation**	
Sac packing	Only the aneurysmal sac is filled with the embolic material
**No parent vessel flow preservation**	
Trapping (sandwich, isolation, and front-to-back-door techniques): with or without sac packing	Embolic materials (coils or plugs) are deployed distally and proximally to the aneurysmal neck to isolate the lesion and to prevent retrograde filling from the collaterals. The outflow artery ‘the back door’ is closed first, followed by inflow artery ‘the front door’.
Inflow occlusion	Occlusion proximal to the aneurysmal neck. It was done when there was no other option to treat the lesion and when angiography confirmed no visible back doors in cases managed by this technique.

**Fig. 5:**
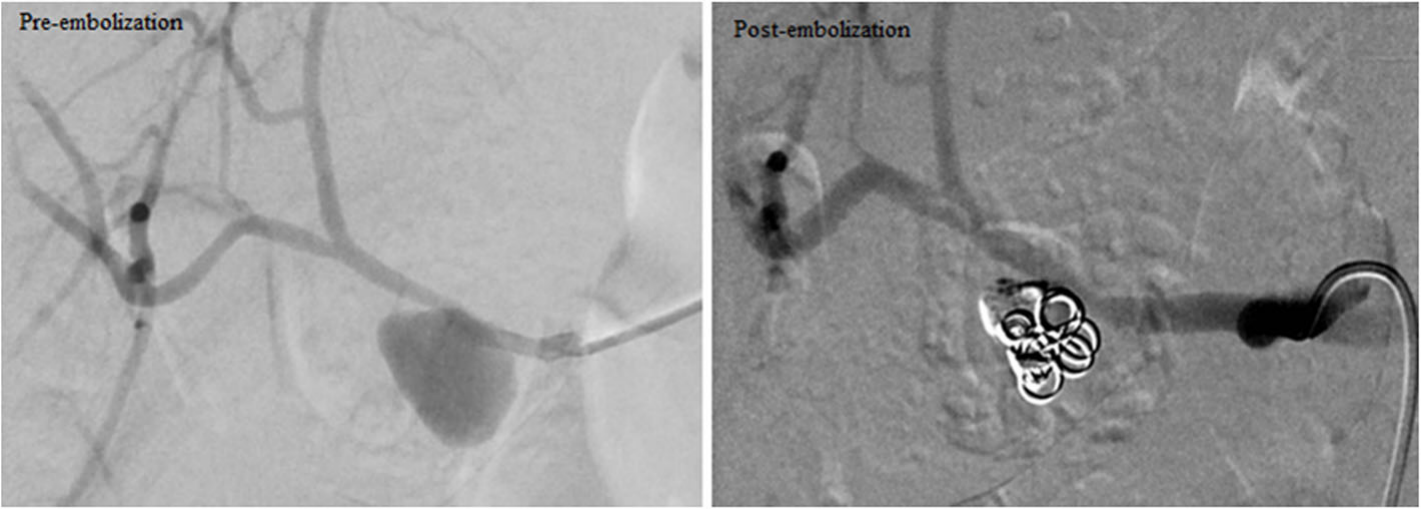
Embolisation of CHA pseudoaneurysm by sac packing technique with multiple coils and NBCA/ Lipiodol mixture

### Follow up

All patients were followed up after discharge for 12 months on an outpatient basis. The follow up protocol of VAPA patients after endovascular treatment consisted of clinical assessment and duplex ultrasound examination at 1, 3, 6, and 12 months. CT was the basic tool of assessment in case of clinical suspicion of complications or symptoms recurrence.

### Study outcomes and definitions

The primary outcomes of this study were:

- Clinical success according to SIR guidelines (Angle et al. 2010): is referred to as the 30-day clinical outcome based on clinical or imaging data or both per established guidelines. Resolution of signs and symptoms that prompted the endovascular procedure along with the absence of unexpected procedure-related complications within 30 days of the endovascular management is considered clinical success.
- Periprocedural complications were classified according to CIRSE classification system (Filippiadis et al. 2017).

The secondary outcomes included:

- Technical success according to SIR guidelines (Angle et al. 2010): is defined as successful deployment of the embolic material within the intended artery with immediate complete aneurysm exclusion in the final angiographic control without evidence of contrast media extravasation.
- Procedure-related 30-day mortality rate.
- Effectiveness of the procedure: depends on complete exclusion of the aneurysm from the circulation without emergence of new symptoms and signs requiring aneurysmal re-intervention during the follow up (Cappucci et al. 2017).
- Target lesion re-intervention rate: is defined as requiring an additional procedure (open surgical or percutaneous or endovascular) due to target lesion recurrence or re-bleeding (Spiliopoulos et al. 2012).

### Statistical analysis

Data was collected and analyzed using SPSS (Statistical Package for the Social Science, version 20, IBM and Armonk, New York). Continuous data were expressed in the form of mean and range while nominal data were expressed in the form of frequency (percentage).

## Results

### Demographics and characteristics of aneurysms among enrolled patients are described in Table 2

**Table 2:** Patients’ demographics and characteristics of the pseudoaneurysms

Demographics	***n*** = 46
Age (Years)	Mean: 58.09 - Range: 21–94
Sex	
- Male	34 (73.9%)
- Female	12 (26.1%)
Risk factors of the vascular lesions:	
- History of previous intervention (either endoscopy, percutaneous needle biopsy or surgery)	26 (56.52%)
- Penetrating duodenal ulcers	7 (15.21%)
- Intrabdominal infection and/ or inflammatory process	7 (15.21%)
- Underlying vascular disease: (Vasculitis)	4 (8.7%)
- Major trauma	1 (2.17%)
- Bleeding colonic diverticula	1 (2.17%)
Presentations	
- GIT hemorrhage and/or haemobilia	16 (34.78%)
- Intra-abdominal hemorrhage	14 (30.43%)
- Hematuria	11 (23.91%)
- Abdominal pain	5 (10.87%)
***Characteristics of aneurysms among enrolled patients***	
Shape of the aneurysm: Saccular	46 (100%)
Mean size of the aneurysm (mm)	Mean: 13 - Range: 2–45
Artery affected:	
- Renal artery	16 (34.78%)
- Gastroduodenal artery	10 (21.74%)
- Superior mesenteric artery	7 (15.22%)
- Hepatic artery	3 (6.52%)
- Pancreaticoduodenal arcades	3 (6.52%)
- Inferior mesenteric artery	3 (6.52%)
- Splenic artery	2 (4.35%)
- Cystic artery	2 (4.35%)
Location of the aneurysm in relation to the segment of the affected artery:	
- Proximal segment	4 (8.7%)
- Middle segment	9 (19.57%)
- Distal segment	33 (71.74%)

### Endovascular management among enrolled patients

Tables 3, 4 & 5 show detailed endovascular management of VAPAs among enrolled patients. Overall clinical success was achieved in 43/46 patients (93.48%). For the subgroup of coils (*n* = 28), clinical success was achieved in 26/28 patients (92.86%). On the other hand, the subgroup of NBCA glue (*n* = 16) showed 93.75% (15/16) clinical success. In lesions managed through sac packing technique (*n* = 9), clinical success was achieved in 7/9 patients (77.78%), while in lesions managed through inflow occlusion (*n* = 28) and trapping techniques (*n* = 9), clinical success was achieved in 27/28 (96.43%), and 9/9 (100%) of the patients, respectively.

**Table 3:** Pathophysiological criteria of the pseudoaneurysms treated with NBCA and their management techniques and outcomes

N	Anatomy	Morphology	Co-Morbidities & risk factors/ Presentation	Urgent or elective management	Embolisation technique	Embolic material	Technical success	Complications	Clinical success	Effectiveness of the procedure	Target lesion Re-intervention
1	SA	23 mm saccular aneurysm	Pancreatitis/ Abdominal pain	Urgent	Sac packing	NBCA Glue	Yes	Grade 2 (Mild post embolisation syndrome)	Yes	Yes	No
2	SMA	45 mm saccular aneurysm	Vasculitis/ Intrabdominal hemorrhage	Urgent	Sac packing	NBCA Glue	Yes	No	Yes	yes	No
3	RT RA	20 mm saccular aneurysm	Iatrogenic (Post pyelolithotomy)/ Hematuria	Urgent	Sac packing	NBCA Glue	Yes	No	Yes	Yes	No
4	RT HA	13 mm saccular aneurysm	Iatrogenic (Post percutaneous needle biopsy)/ Intrabdominal hemorrhage	Urgent	Sac packing	NBCA Glue	Yes	No	Yes	Yes	No
5	LT RA	5.5 mm saccular aneurysm	Iatrogenic (Post percutaneous needle biopsy)/ Hematuria	Urgent	Sac packing	NBCA Glue	Yes	No	Yes	Yes	No
6	Cystic a	20 mm saccular aneurysm	Acute cholecystitis/ GIT bleeding and haemobilia	Urgent	Sac packing	NBCA Glue	Yes	Grade 3 (Ischemia of the GB with subsequent necrosis & abscess formation)	No	Yes	No
7	GDA	8 mm saccular aneurysm	Penetrating duodenal ulcer/ GIT bleeding	Urgent	Inflow occlusion	NBCA Glue	Yes	No	Yes	Yes	No
8	RT RA	11 mm saccular aneurysm	Iatrogenic (Post percutaneous needle biopsy)/ Hematuria	Urgent	Inflow occlusion	NBCA Glue	Yes	Grade 2 (Mild post embolisation syndrome)	Yes	Yes	No
9	LT RA	2 mm saccular aneurysm	Vasculitis/ Intrabdominal hemorrhage	Urgent	Inflow occlusion	NBCA Glue	Yes	No	Yes	Yes	No
10	GDA	2.5 mm	Vasculitis/ GIT bleeding	Urgent	Inflow occlusion	NBCA Glue	Yes	No	Yes	Yes	No
11	RT RA	4 mm saccular aneurysm	Iatrogenic (Post pyelolithotomy)/ Hematuria	Urgent	Inflow occlusion	NBCA Glue	Yes	No	Yes	Yes	No
12	LT RA	9 mm saccular aneurysm	Iatrogenic (Post pyelolithotomy)/ Hematuria	Urgent	Inflow occlusion	NBCA Glue	Yes	No	Yes	Yes	No
13	LT RA	5.3 mm saccular aneurysm	Iatrogenic (Post percutaneous needle biopsy)/ Intrabdominal hemorrhage	Urgent	Inflow occlusion	NBCA Glue	Yes	No	Yes	Yes	No
14	RT HA	13 mm saccular aneurysm	Iatrogenic (Post percutaneous needle biopsy)/ Intrabdominal hemorrhage	Urgent	Inflow occlusion	NBCA Glue	Yes	No	Yes	Yes	No
15	RT RA	24 mm saccular aneurysm	Iatrogenic (Post percutaneous needle biopsy)/ Hematuria	Urgent	Inflow occlusion	NBCA Glue	Yes	No	Yes	Yes	No
16	GDA	13 mm saccular aneurysm	Penetrating duodenal ulcer/ GIT bleeding	Urgent	Inflow occlusion	NBCA Glue	Yes	No	Yes	Yes	No

*SA* splenic artery, *SMA* superior mesenteric artery, *RA* renal artery, *HA* hepatic artery, *GDA* gastroduodenal artery, *NBCA* N-butylcyanoacrylate

**Table 4:** Pathophysiological criteria of the pseudoaneurysms treated with coils and their management techniques and outcomes

N	Anatomy	Morphology	Co-Morbidities & risk factors/ Presentation	Urgent or elective management	Embolisation technique	Embolic material	Technical success	Complications	Clinical success	Effectiveness of the procedure	Target lesion Re-intervention
1	GDA	44.5 mm Saccular aneurysm	Iatrogenic (Post percutaneous needle biopsy)/ Abdominal pain	Urgent	Trapping	3 detachable micro coils	Yes	No	Yes	Yes	No
2	GDA	2.5 mm saccular aneurysm	Penetrating duodenal ulcer/ GIT bleeding	Urgent	Sac packing	2 pushable coils	Yes	No	Yes	Yes	No
3	GDA	10.5 mm saccular aneurysm	Pancreatitis/ Intrabdominal hemorrhage	Urgent	Trapping with sac packing	3 detachable micro coils	Yes	No	Yes	Yes	No
4	GDA	26.5 mm saccular aneurysm	Penetrating duodenal ulcer/ GIT bleeding	Urgent	Trapping with occlusion of the collaterals	3 detachable micro coils	Yes	No	Yes	Yes	No
5	IMA	14.5 mm saccular aneurysm	Iatrogenic (Post lumbar discectomy)/ GIT bleeding	Urgent	Trapping	2 detachable micro coils	Yes	No	Yes	Yes	No
6	GDA	15 mm saccular aneurysm	Penetrating duodenal ulcer/ GIT bleeding	Urgent	Trapping	2 detachable micro coils	Yes	No	Yes	Yes	No
7	GDA	15 mm saccular aneurysm	Pancreatitis/ Abdominal pain	Urgent	Trapping	3 detachable micro coils	Yes	No	Yes	Yes	No
8	Pancreaticoduodenal a	3 mm saccular aneurysm	Iatrogenic (Post percutaneous needle biopsy)/ Intrabdominal hemorrhage	Urgent	Trapping	3 detachable micro coils	Yes	No	Yes	Yes	No
9	IMA	4 mm saccular aneurysm	Iatrogenic (Post colonscopic polypectomy) / GIT bleeding	Urgent	Trapping with sac packing	3 detachable micro coils	Yes	No	Yes	Yes	No
10	Cystic a	7 mm saccular aneurysm	Iatrogenic (Post ERCP)/ GIT bleeding and haemobilia	Urgent	Sac packing	Single detachable micro coil	Yes	Grade 3 (Re-bleeding required re-intervention)	No	No (Re-filling of the aneurysmal sac on follow up)	Yes (Sac packing then inflow occlusion by NBCA)
11	RT RA	3 mm saccular aneurysm	Iatrogenic (Post pyelolithotomy)/ Hematuria	Urgent	Inflow occlusion	Single pushable coil	Yes	No	Yes	Yes	No
12	LT RA	20 mm saccular aneurysm	Iatrogenic (Post pyelolithotomy)/ Hematuria	Urgent	Inflow occlusion	2 pushable coils	Yes	Grade 2 (Mild post embolisation syndrome)	Yes	Yes	No
13	Pancreaticoduodenal a	27 mm saccular aneurysm	Penetrating duodenal ulcer/ Intrabdominal hemorrhage	Urgent	Inflow occlusion	2 detachable micro coils	Yes	No	Yes	Yes	No
14	SMA	34 mm saccular aneurysm	Vasculitis/ Intraabdominal hemorrhage	Urgent	inflow occlusion	3 detachable micro coils	Yes	No	Yes	Yes	No
15	Pancreaticoduodenal a	19.75 mm saccular aneurysm	Penetrating duodenal ulcer/ Intrabdominal hemorrhage	Urgent	Inflow occlusion	2 detachable micro coils	Yes	No	Yes	Yes	No
16	RT RA	5.5 mm saccular aneurysm	Iatrogenic (Post percutaneous needle biopsy)/ Hematuria	Urgent	Inflow occlusion	2 pushable coils	Yes	No	Yes	Yes	No
17	RT RA	5 mm saccular aneurysm	Iatrogenic (post percutaneous needle biopsy)/ Hematuria	Urgent	Inflow occlusion	Single pushable coil	Yes	No	Yes	Yes	No
18	LT RA	14.1 mm saccular aneurysm	Iatrogenic (Post percutaneous needle biopsy)/ Intrabdominal hemorrhage	Urgent	Inflow occlusion	2 pushable coils	Yes	No	Yes	Yes	No
19	RT RA	3 mm saccular aneurysm	Iatrogenic (Post pyelolithotomy)/ Hematuria	Urgent	Inflow occlusion	2 pushable coils	Yes	No	Yes	Yes	No
20	RT RA	3.5 mm saccular aneurysm	Iatrogenic (Post percutaneous needle biopsy)/ Intrabdominal hemorrhage	Urgent	Inflow occlusion	2 detachable micro coils	Yes	No	Yes	Yes	No
21	LT RA	33.5 mm saccular aneurysm	Septic emboli/ Abdominal pain	Urgent	Inflow occlusion	3 detachable micro coils	Yes	No	Yes	Yes	No
22	SA	3.5 mm saccular aneurysm	Trauma/ Intrabdominal hemorrhage	Urgent	Inflow occlusion	2 pushable micro coils	Yes	No	Yes	Yes	No
23	SMA	2 mm saccular aneurysm	Iatrogenic (Post inguinal hernia repair)/ GIT bleeding	Urgent	Inflow occlusion	2 detachable micro coils	Yes	Grade 4 (Bowel loop ischemia)	No	Yes	No
24	SMA	5 mm saccular aneurysm	Iatrogenic (Post colonscopic polypectomy)/ GIT bleeding	Urgent	Inflow occlusion	3 detachable micro coils	Yes	Grade 2 (Mild post embolisation syndrome)	Yes	Yes	No
25	SMA	2.2 mm saccular aneurysm	Iatrogenic (Post colonscopic polypectomy)/ GIT bleeding	Urgent	Inflow occlusion	2 detachable micro coils	Yes	No	Yes	Yes	No
26	SMA	3.2 mm saccular aneurysm	Iatrogenic (Post colonscopic polypectomy)/ GIT bleeding	Urgent	Inflow occlusion	2 detachable micro coils	Yes	No	Yes	Yes	No
27	SMA	5.1 mm saccular aneurysm	Iatrogenic (Post colonscopic polypectomy)/ GIT bleeding	Urgent	Inflow occlusion	2 detachable micro coils	Yes	No	Yes	Yes	No
28	IMA	5 mm saccular aneurysm	Diverticula / GIT bleeding	Urgent	Inflow occlusion	2 detachable micro coils	Yes	No	Yes	Yes	No

*GDA* gastroduodenal artery, *IMA* inferior mesenteric artery, *RA* renal artery, *SMA* superior mesenteric artery, *SA* splenic artery

**Table 5:** Pathophysiological criteria of the pseudoaneurysms treated with Amplatzer vascular plugs or mixed NBCA & coils and their management techniques and outcomes

N	Anatomy	Morphology	Co-Morbidities & risk factors/ Presentation	Urgent or elective management	Embolisation technique	Embolic material	Technical success	Complications	Clinical success	Effectiveness of the procedure	Target lesion Re-intervention
1	CHA **(**Fig. 5**)**	30 mm saccular aneurysm	Infection post whipple/ Abdominal pain	Urgent	Sac packing	4 pushable coils and NBCA glue	Yes	No	Yes	Yes	No
2	GDA	13 mm saccular aneurysm	Pancreatitis/ Intrabdominal hemorrhage	Urgent	Trapping	6.5 mm and 5 mm diameter microvascular plugs & 7 mm diameter Amplatzer IV plug	Yes	No	Yes	Yes	No

*CHA* common hepatic artery, *GDA* gastroduodenal artery, *NBCA* N-butylcyanoacrylate

Periprocedural complications were reported in 7/46 patients (15.22%). Grade-2 complication was reported in 4 patients (8.7%) representing mild post embolisation syndrome (transient pain requiring only oral analgesia with no prolongation of hospital stay). Grade-3 complication was reported in 1 patient (2.17%) that had cystic artery pseudoaneurysm and was complicated by aneurysmal sac rupture and re-bleeding after being managed by coils through sac packing technique. That was successfully managed by inflow occlusion of the parent artery using NBCA glue. Grade-4 complication (permanent mild sequelae) was reported in two patients (4.35%). One patient with cystic artery pseudoaneurysm after being embolised by NBCA glue through sac packing technique developed ischemia of the gall bladder with subsequent necrosis and abscess formation that required further percutaneous tubal drainage and cholecystectomy later-on. The other patient had pseudoaneurysm in the jejunal branch of SMA and was complicated by focal jejunal loop ischemia after being managed by coils through inflow occlusion technique. That was successfully managed by laparotomy and resection anastomosis surgery of the ischemic jejunal loop. The 3 patients who had grade 3 & 4 complications were responsible for the small percentage of the overall clinical failure in the study.

Technical success was achieved in 100% of the patients with no reported 30-day mortality in the study. Procedure effectiveness was achieved in 45/46 patients (97.83%). Only one patient required re-intervention that had cystic artery pseudoaneurysm with successful clinical outcome later-on.

## Discussion

It is essential to mention that the clinical response of endovascular embolisation of VAPA depends on the type of the embolic agent and adequacy of the embolisation process. When choosing an embolic agent, many factors should be taken into consideration. These factors include site, and size of the lesion, as well as the flow pattern of vessels to be occluded, the availability of embolic agents, the experience and knowledge of the radiologist who will perform the procedure, the speed and reliability of delivery, the duration of the occlusive effect, and the avoidance of non-target embolisation (Ząbkowski et al. 2015). In this study, mainly permanent occlusive agents were used to avoid recanalization of the lesion and recurrence of presenting symptoms would be expected to be less. Coils were the most frequent materials used in the management either alone (60.87%) or with NBCA glue (2.17%). Embolisation techniques used in the study were sac packing, inflow occlusion and trapping in 19.57%, 60.87% and 19.57% of the patients, respectively.

In this study, the overall clinical success rate was 93.48% with zero 30-day mortality rate. These results were comparable to those of Venturini et al. who achieved 83% clinical success with a 7% 30-day mortality rate (Venturini et al. 2017). For the subgroup of coils (*n* = 28), the clinical success was 92.86%, while the subgroup of NBCA glue (*n* = 16) showed clinical success of 93.75%. These results were similar to Alwarraky et al. who reported a clinical success of 91.3% and 93.3% in lesions embolised with coils and NBCA, respectively in the endovascular management of acute renal bleeding (Alwarraky et al. 2020).

In the current study, 7/46 patients (15.22%) developed periprocedural complications. Although inconsistencies in reporting complications among studies in the literature were noted, including small visceral infarcts detected on follow-up imaging accounting for the wide range of reported complication rates (0–50%), the complication rate of 15.22% reported in this study was comparable to the overall composite complication rate of 18.2% (Kilani et al. 2016; Zhang et al. 2016; Patel et al. 2012; Kok et al. 2016). CIRSE Quality Assurance Document and Standards for Classification of Complications was used in this study to eliminate subjective interpretation of adverse event.

**Fig. 6:**
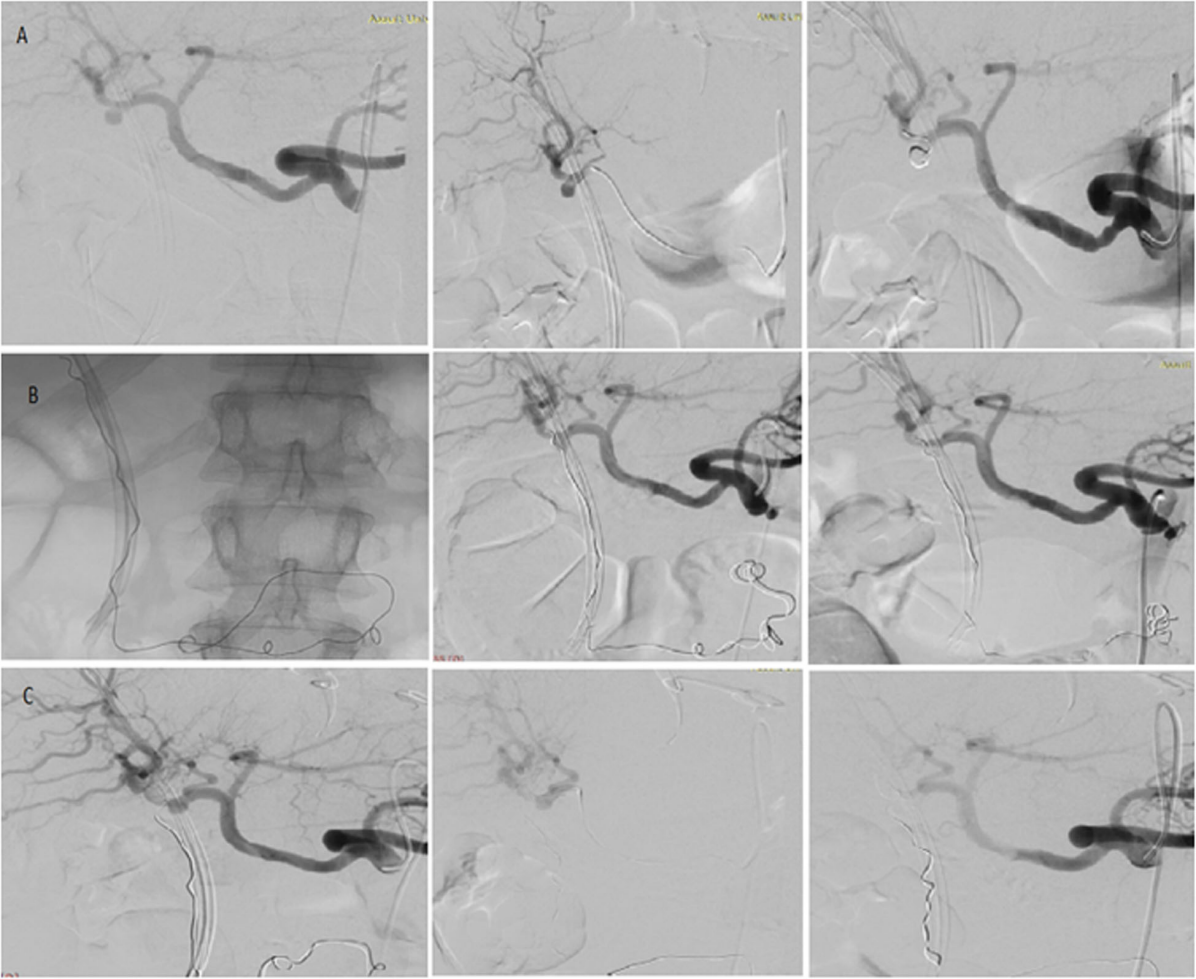
Embolisation of cystic artery pseudoaneurysm. **a** Coiling of cystic artery pseudoaneurysm using sac packing technique. **b** Embolisation of the re-filled pseudoaneurysm using NBCA/ Lipiodol mixture ‘sac packing technique’. **c** Embolisation of right hepatic artery proximal to the stump of cystic artery after 2nd time re-filling of the pseudoaneurysm using NBCA/ Lipiodol mixture ‘inflow occlusion’

In this study, technical success rate was 100%, and this was comparable to the most of other similar studies in the literature (Khattak et al. 2014; Madhusudhan et al. 2015; Won et al. 2015; Fankhauser et al. 2011). Procedure effectiveness in the current study was 97.83% with complete aneurysmal sac exclusion without the emergence of new symptoms and signs requiring aneurysmal re-intervention. Only one patient with a cystic artery aneurysm showed revascularization of the aneurysmal sac on follow up imaging. In line with this result, Spiliopoulos et al. that showed a long-term efficacy of endovascular management with only 6.1% target lesion re-intervention rate during a mean period of follow-up of 19.1 ± 21.4 months (Spiliopoulos et al. 2012). In this series, the target lesion re-intervention rate was 2.17% (the patient who had a cystic artery pseudoaneurysm). The pseudoaneurysm was initially embolised by 30 cm × 6 mm detachable micro coil. However, it was complicated after 1 week of the procedure by rupture of the aneurysmal sac and migration of the coil into the CBD down the duodenum (Fig. 6); hence, re-embolisation was done using NBCA in two different sessions; in the first session, sac packing was done with complete aneurysm exclusion from the final angiographic image. Again, it was complicated by sac rupture 1 month later. In the second session, parent artery embolisation (inflow occlusion) was done successfully. Target lesion re-intervention rate in previous studies ranged between 6.7–15% (Pitton et al. 2015; Venturini et al. 2017; Spiliopoulos et al. 2012).

The satisfactory results of endovascular embolisation could be due to the continuous advances in embolic materials and catheter designs used in interventional catheter-based techniques; the development of microcatheter technology has enabled selective catheterization of even small-caliber vessels and the use of micro coils and different polymerization rates of NBCA glue has allowed a more targeted embolisation (Venturini et al. 2017).

The main limitations in this study were 1) the retrospective design of the study 2) the mid-term evaluation and so, knowledge of the durability of embolisation is limited to 1 year only, and 3) the non-randomization of the studied subgroups. In the future, a randomized prospective study to compare efficacy of each embolic agent and each embolisation technique is desirable.

## Conclusion

Transarterial embolisation of visceral artery pseudoaneurysms can provide high technical and clinical success rates with low periprocedural complication and re-intervention rates, as well as satisfactory procedure effectiveness in the management of VAPAs.

## Data Availability

The datasets used and/or analyzed during the current study are available from the corresponding author on reasonable request.

## Abbreviations

VAA: Visceral artery aneurysm
VAPA: Visceral artery pseudoaneurysm
GIT: Gastrointestinal
CT: Computed tomography
NBCA: N-butylcyanoacrylate
SIR: Society of Interventional Radiology
CIRSE: Cardiovascular and interventional radiological society of Europe
SMA: Superior mesenteric artery
IMA: Inferior mesenteric artery
CHA: Common hepatic artery
HA: Hepatic artery
RA: Renal artery
SA: Splenic artery
GDA: Gastroduodenal artery
